# Access to medicines for treating people with cryptococcal meningitis

**DOI:** 10.1093/cid/ciac689/6678599

**Published:** 2023-02-08

**Authors:** Jessica Burry, Carmen Perez Casas, Nathan Ford

**Affiliations:** 1 MSF Access Campaign, Geneva, Switzerland; 2 Unitaid, Geneva, Switzerland; 3 Dept HIV, Viral Hepatitis and STIs, World Health Organization, Geneva, Switzerland

**Keywords:** Acess, cryptococcal meningitis, HIV/AIDS, liposomal amphotericin B, mortality

## Abstract

Cryptococcal meningitis accounts for one in five AIDS-related deaths globally. WHO guidelines strongly recommend a single high-dose of liposomal amphotericin B as part of preferred treatment, but this drug remains unaffordable in most low- and middle-income countries. A proactive approach is needed from manufacturers and other stakeholders to improve access.

The decline in HIV-associated deaths has stagnated, and there is a growing appreciation that advanced HIV disease is a persistent challenge that requires targeted attention. Cryptococcal meningitis remains a leading cause of HIV-associated mortality(1). An updated review of the global burden of cryptococcal disease shows little change over the past decade. From 2020 estimates, there are 179,000 cases of cryptococcal antigenemia (infection) globally in 2020 and 152,000 cases of cryptococcal meningitis, resulting in 112,000 cryptococcal-related deaths. There has been a reduction in the estimated absolute global burden of HIV-associated cryptococcal meningitis compared to estimates from 2014, likely due to expanded coverage of antiretroviral therapy; however, cryptococcal disease still accounts for one in five AIDS-related deaths, similar to previous estimates (1, 2).

The new clinical management of cryptococcal meningitis guidelines issued by the World Health Organization (WHO) in June 2022 strongly recommend a single high-dose of liposomal amphotericin B with 2 weeks of flucytosine and fluconazole as the preferred treatment (3). This recommendation was made following a multi-country trial showing this regimen is at least as effective as the standard of care, with better safety, and fewer monitoring demands. The favourable safety profile of the single-dose liposomal amphotericin B–containing regimen has a lower risk of anaemia and hypokalaemia, reducing the intensity of monitoring and the need to manage drug-related toxicity(4). The single high-dose containing regimen was also preferred by providers because it was less time consuming to prepare, and may allow for faster hospital discharge (5).

Liposomal amphotericin B is a preferred drug for the management of a number of co-infections that are common among people living with HIV. In the last two years WHO has released guidelines for the management of disseminated histoplasmosis(6) and visceral leishmaniasis(7) in people living with HIV and both of these guidelines recommend liposomal amphotericin B as part of a preferred treatment regimen. However, despite having received regulatory approval in 1997(8), and being off patent since 2016 in the United States(9), the drug remains unaffordable and unavailable in public healthcare systems in most low- and middle-income countries (LMICs).

In 2018 the brand manufacturer, Gilead, committed with Unitaid to a preferential price of $16.25 per vial for 116 countries but, as of the end of 2021, less than half of eligible countries had been able to procure at this preferential price. Those that have been able to access this no-profit price have often faced long lead-times, supply shortages and procurement delays. Meanwhile, Gilead has recently indicated that it intends to increase the no-profit price by almost 25% to $19-20 per vial in early 2023.

In South Africa, a country with a high burden of HIV and consequently mortality due to cryptococcal meningitis, liposomal amphotericin B is still priced as high as $205 per vial or $2,500 per person(10) in the private market while not even available in the public sector. In India, it is available only in the private market via Gilead’s distributor, Viatris, at a price of $69 per vial. In Brazil, where Gilead’s preferential pricing is applicable for treatment of leishmaniasis, but not for cryptococcal meningitis, the price reaches $373 USD per vial or approximately $4,500 USD to treat one person (11). Access to this preferential pricing is limited mainly to cryptococcal meningitis.

Figure 1 illustrates the disparity of price of a 50 mg vial of liposomal amphotericin B compared to country wealth (as measured by gross national income): low and middle-income countries with a high burden of HIV - including Brazil, Peru, South Africa, and Thailand – are paying far more than high-income countries in Europe such as Belgium, France, Luxembourg, Spain and Switzerland.

A new pathway for more sustainable access is opening, with some generic manufacturers developing liposomal amphotericin B, and one generic already approved by the US FDA (16 December 2021). UNITAID has issued an expression of interest(12) to offer support in overcoming barriers to market entry, in an effort to ensure the successes seen in these landmark clinical trials translate into broader access. While demand to date has been low, this could change with the adoption of the new guidelines for managing cryptococcal disease and improved access conditions.

While the lack of patent barrier means generics manufacturers are free to enter the global market, they are more likely to target commercially lucrative markets of high-income or upper-middleincome countries, where the originator drug is sold at excessively high prices the United States for example, where liposomal amphotericin B from Gilead sells at more than $300 per vial, the market is worth approximately $136 million annually (13).

Generic companies must allocate a portion of their manufacturing capacity to supplying LMICs at an affordable price, many of which still do not have adequate access to this life-saving medicine and cannot provide the gold standard of treatment for people with cryptococcal meningitis.

Flucytosine, first approved by the USFDA in 1971, is an essential component in the treatment regimen for cryptococcal meningitis, however it too is not found in most high-burden countries. Since 2017, five new generic sources have been approved’by the USFDA or WHO PQ, however this has not translated into increased access in LMICs. Insufficient testing for cryptococcal meningitis and lack of inclusion in national cryptococcal meningitis treatment guidelines has led to an unclear market demand, which, in addition to unclarity on funding does not incentivize companies to register and supply broadly.

Lack of registration is another barrier (10). Liposomal amphotericin B from the originator is registered for use in few LMICS, and only two countries in sub-Saharan Africa (10). Access to flucytosine is also limited because, among other reasons, it is not registered in the majority of countries in Africa. Despite being big players in the HIV market, two of the generic manufacturers of flucytosine have registered only in the United States but do not supply it more broadly in countries where it is needed for cryptococcal meningitis.

Twenty years ago, Mèdecins Sans Frontieres (MSF) highlighted inequities in access to another drug to prevent and treat cryptococcal meningitis, fluconazole, due to its high price, lack of competition and irrational pricing (14). WHO’s new guidelines for cryptococcal meningitis will only translate into lives saved if a proactive approach is taken by the manufacturers and other stakeholders, together with national governments and the international community, to solving these challenges.

## Figures and Tables

**Figure 1:**
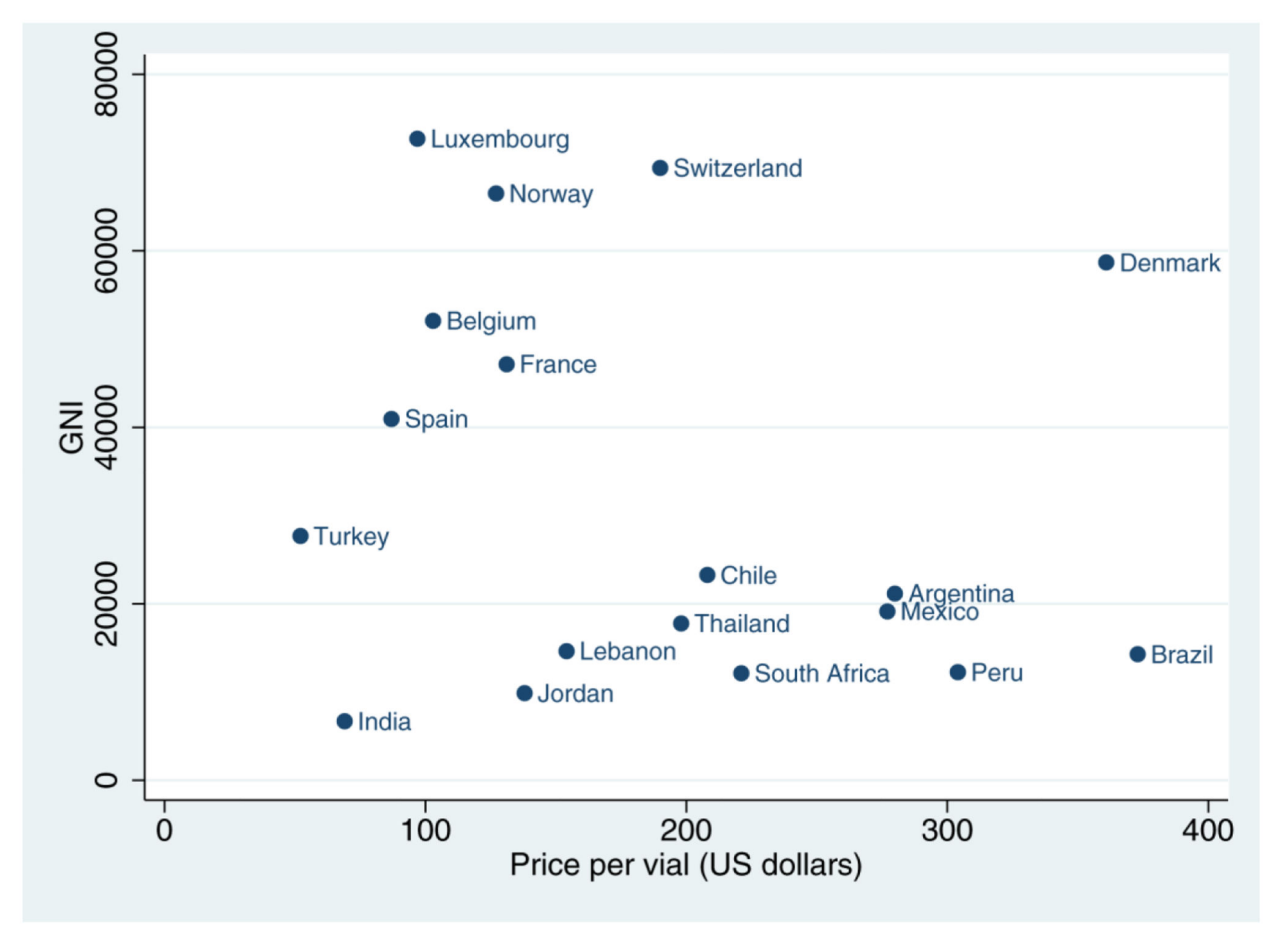
Correlation between price of liposomal amphotericin B and gross national income Full pricing table and sources in Supplementary Table 1. Data on gross national income ($US) obtained from the United Nations Development Programme.(15)
